# Cell adhesion molecule BVES functions as a suppressor of tumor cells extrusion in hepatocellular carcinoma metastasis

**DOI:** 10.1186/s12964-022-00962-9

**Published:** 2022-09-19

**Authors:** Ping Han, Yu Lei, Jingmei Liu, Jiqiao Liu, Huanjun Huang, Dean Tian, Wei Yan

**Affiliations:** 1 Department of Gastroenterology, Tongji Hospital of Tongji Medical College, Huazhong University of Science and Technology, Wuhan, 430030 Hubei Province China; 2 Department of Ultrasound, Tongji Hospital of Tongji Medical College, Huazhong University of Science and Technology, Wuhan, 430030 Hubei Province China

**Keywords:** Hepatocellular carcinoma, Extrusion, BVES, Metastasis, Rho

## Abstract

**Background:**

Tumor cells detachment from primary lesions is an early event for hepatocellular carcinoma (HCC) metastasis, in which cell adhesion molecules play an important role. The role of mechanical crowding has attracted increasing attention. Previous studies have found that overcrowding can induce live cells extrusion to maintain epithelial cell homeostasis, and normally, live extruded cells eventually die through a process termed anoikis, suggesting the potential of tumor cells resistant to anoikis might initiate metastasis from primary tumors by cell extrusion. We have demonstrated transmembrane adhesion molecule blood vessel epicardial substance (BVES) suppression as an early event in HCC metastasis. However, whether its suppression is involved in HCC cell extrusion, especially in HCC metastasis, remains unknown. This study aims to investigate the role of BVES in tumor cells extrusion in HCC metastasis, as well as the underlying mechanisms.

**Methods:**

Cells extrusion was observed by silicone chamber, petri dish inversion, and three-dimensional cell culture model. Polymerase chain reaction, western blotting, immunohistochemistry, immunofluorescence, co-immunoprecipitation, and RhoA activity assays were used to explore the underlying mechanisms of cell extrusion regulated by BVES. An orthotopic xenograft model was established to investigate the effects of BVES and cell extrusion in HCC metastasis in vivo.

**Results:**

Tumor cell extrusion was observed in HCC cells and tissues. BVES expression was decreased both in HCC and extruded tumor cells. BVES overexpression led to the decrease in HCC cells extrusion in vitro and in vivo. Moreover, our data showed that BVES co-localized with ZO-1 and GEFT, regulating ZO-1 expression and localization, and GEFT distribution, thus modulating RhoA activity.

**Conclusion:**

The present study revealed that BVES downregulation in HCC enhanced tumor cells extrusion, thus promoting HCC metastasis, which contributed to a more comprehensive understanding of tumor metastasis, and provided clues for developing novel HCC therapy strategies.

**Video abstract**

**Supplementary Information:**

The online version contains supplementary material available at 10.1186/s12964-022-00962-9.

## Background

Hepatocellular carcinoma (HCC) is one of the most common cancers worldwide with a high mortality rate [1]. Early intrahepatic metastasis and postoperative recurrence and metastasis are the main causes of poor prognosis of HCC patients [2]. The metastatic process of HCC involves several stages, including tumor cells detachment from the primary lesions, infiltration of the extracellular matrix layer and vascular system, and adhesion infiltration into the target organs to colonize and proliferate to form metastases [3]. Therefore, exploring the ways and molecular mechanisms of tumor cell detachment from primary lesions might provide new clues for preventing tumor metastasis of HCC.

Cells detachment is an early event for tumor cell metastasis, and cell adhesion molecules play an important role in this process. Mechanical crowding and stretch-activated signaling play a vital role in epithelial cell homeostasis [4]. Normally, a doomed cell triggers the formation of actin-myosin rings in the surrounding live neighboring epithelial cells, extruding the dying cell out to maintain epithelial barrier function [5]. Recent studies have revealed a novel role of extrusion in controlling developmental morphogenesis and maintaining cell number homeostasis. In crowded regions of the tissue, a proportion of cells experience a sustained loss of cell–cell junctions, while crowding induces neighboring cells to activate Rho-mediated assembly and contraction of intercellular actin-myosin rings, ultimately extruding the excessive live cells [6, 7]. In normal situations, the extruded live cells eventually die through a process known as anoikis, or apoptosis due to loss of survival signaling [8, 9]. Blood vessel epicardial substance (BVES), also known as Popeye domain-containing protein 1 (POPDC1), is firstly described as a three-pass transmembrane protein that localizes to the plasma membrane [10]. Previous studies have shown that BVES co-localizes with zonula occludens-1 (ZO-1) and occludin in polarized epithelial cells for modulation of epithelial cell integrity [11, 12]. However, whether the cell transmembrane adhesion molecule BVES is involved in live cell extrusion remains elusive to date. It has been reported that BVES regulates RhoA activation in trabecular meshwork (TM) cells, and overexpression of BVES lead to the increased formation of tight junction (TJ), which regulates Rho activation by sequestering guanine nucleotide exchange factor (GEF) with BVES/ZO-1 complex in the cell membrane [13, 14]. We suppose that BVES may regulate the extrusion of live cells through Rho GTPases signaling pathway.

In the present study, we found that the extrusion of tumor cells was frequently observed in HCC cells and tissues, which was closely associated with HCC metastasis. Furthermore, the results also demonstrated that BVES regulated the extrusion of HCC cells. Mechanistically, the BVES/ZO-1/Rho GTPases pathway might be an important regulator of HCC cell extrusion.

## Methods and materials

### Cell culture

The HCC cell lines were kept in the Institute of Liver and Gastrointestinal Diseases (Tongji Hospital, Huazhong University of Science and Technology). HepG2, Huh7, SMMC-7721, MHCC97L, MHCC97H, HCCLM3, and SK-hep-1 were cultured in DMEM medium, HL7702 were cultured in RPMI 1640 medium, and Hep3B were cultured in MEM medium. These cell lines were incubated in at 37 °C, 5% CO2. All of the medium were added 10% fetal bovine serum (Invitrogen Gibco, USA).

### Human samples

The human HCC and adjacent nontumor tissues were obtained from patients who underwent excisional surgery of HCC in Tongji hospital. The patients received no preoperative adjuvant therapy. The diagnosis of HCC was confirmed pathologically. This study was approved by the Ethics Committee of Tongji Hospital and complied with the World Medical Association Declaration of Helsinki.

### In vitro silicone chamber model

**Fig. 1:**
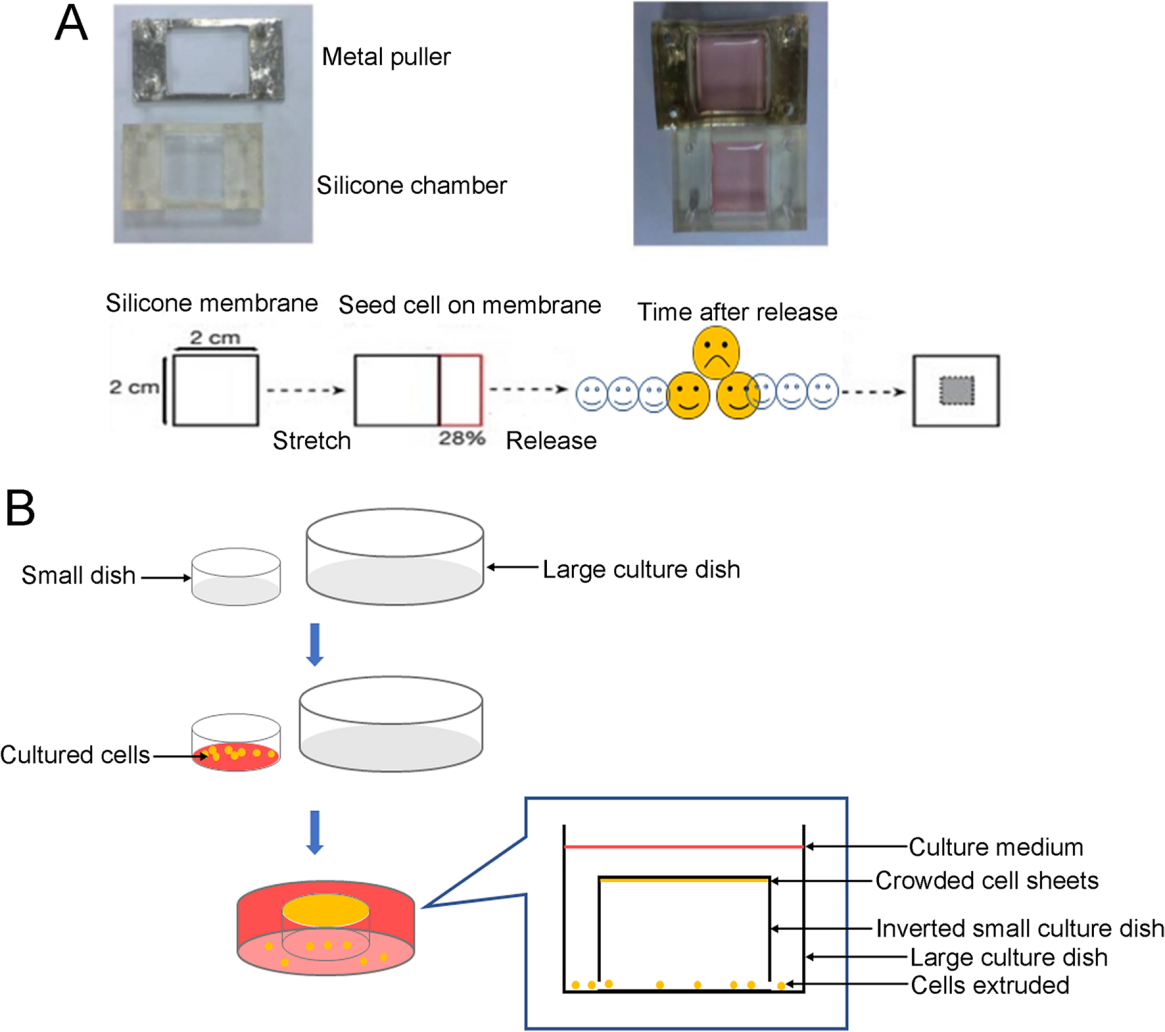
In vitro silicone chamber model and petri dish inversion model. **A** The schematic diagram of silicone chamber model. **B** The schematic diagram of petri dish inversion model

A silicone membrane stretching model for detecting live cell extrusion was invented by Professor Jody Rosenblatt [6]. Briefly, silicone chambers (2 cm × 2 cm) were pre-stretched by 28% through a metal retractor plate (a layer of extracellular matrix adhesive was placed on the silicone membrane to facilitate cell adhesion), and then treated at 4 °C for 24 h. Approximately 750,000 cells were seeded on the stretched silicone membrane. When the cells were fully fused, the silicone chambers were slowly removed to instantly reduce the cell growth area and provide a state of cell overcrowding. Two hours later, the extruded cells were collected from the culture medium, centrifuged and resuspended, and the number of extruded cells was counted (Fig. 1A).

### In vitro petri dish inversion model

We have established an in vitro petri dish inversion model to detect crowding-induced cell extrusion [15]. As shown in Fig. 1B, cells grown to approximately 100% confluence in a small culture dish (60 × 15 mm) were inverted in a large culture dish (150 mm × 25 mm) with enough culture medium to ensure that the medium in the large dish could cover the bottom of the small dish. The gas in the small dish was drawn out using an aseptic bending needle. Live cells extracted from the small culture dish could live in the large culture dish below. Twenty-four hours later, the small culture dish was gently removed. The cells shed from the small dish were able to grow for 7 days. For BVES expression analysis, HCC cells in the large culture dish were collected, and the expression level of BVES in the extruded HCC cells was detected by RT-qPCR as described below. For cell counting analysis, HCC cells in the large culture dish were then stained with crystal violet to detect the formation of cell clones. All steps were aseptic, and touching the bottom of the small dish was also avoided.

### Immunofluorescence (IF) staining

For IF assays, cells were fixed with 4% paraformaldehyde at room temperature for 15 min and, then, permeabilized with phosphate-buffered saline (PBS) containing 0.3% Triton X-100 for 10 min, blocked with 10% goat serum for 40 min, and incubated with primary antibodies at 4 °C overnight. Primary antibodies include GM130 (Abcam, cat#ab52649), BVES (Santa cruz, cat#sc-374081), ZO-1 (GenTex, cat#GTX108592), F-actin (Keygene Biotech, cat#KGMP001 and cat#KGMP0012), GEFT (Proteintech, cat#14839-1-AP). The cells were washed with cold PBS and then incubated with the appropriate secondary antibodies (Promoter, Wuhan, China). Fluorescence was detected using an Olympus fluorescence microscope. As for confocal microscope analysis, images were obtained under a laser confocal microscope or reconstructed by continuous multilayer scanning with a layer spacing of 1.0 mm by the FV10-ASW 2.1 Viewer software.

### Three-dimensional (3D) cell culture

The procedure of 3D cell culture was performed as described previously [16]. In short, 1 × 10^5^ cells were resuspended in 120 μl of 10% sucrose solution, and were quickly mixed with an equal volume of 0.5% hydrogel solution. Then, the cells were immediately seeded in a glass-bottomed cell culture dish. The medium was changed every other day. The cells were allowed to grow for several days and then imaged by microscopy or immunofluorescently stained.

### Immunohistochemistry (IHC) assays

As we previously described [17], the tissues were paraformaldehyde-fixed and paraffin-embedded, and the SP Detection System Kits were used for immunohistochemistry staining (ZSGB-BIO, cat# SP-9000) according to the instructions. The intensity of staining was divided into 0 (negative), 1 (weak), 2 (medium) and 3 (strong). The percentage of positive cells was scored from 0 to 4 (0%, 1–25%, 26–50%, 51–75%, 76–100%, respectively). Total score ranged from 0 to 12.

### Western blot analyses

The protein of HCC cell lines was digested in RIPA buffer containing phosphatase inhibitor cocktail, and PMSF (Wuhan Sevier Biotechnology Co., Ltd.). All samples were centrifuged at 12,000 × *g* for 15 min after grinding and sonication. The protein concentration was determined with BCA Protein Assay Kit (Wuhan Sevier Biotechnology Co., Ltd.). Then, 40 μg protein of each sample was fractionated by SDS-PAGE and transferred to nitrocellulose membranes. Nonspecific binding sites were blocked with 5% milk in TBST (120 mM Tris–HCl (pH 7.4), 150 mM NaCl, and 0.05% Tween 20) for 1 h at room temperature. Blots were incubated with the specific antibody overnight at 4 °C. Primary antibodies include BVES (Santa cruz, cat#sc-374081), ZO-1 (GenTex, cat#GTX108592), GEFT (Proteintech, cat#14839-1-AP). Anti-GAPDH (Proteintech, cat#60004-1-Ig) was used as an internal control. The membranes were then washed with PBS three times and incubated with the appropriate HRP-conjugated secondary antibodies. Immune complexes were visualized using Super ECL Detection Reagent (Yeasen, 36208ES60).

### Quantitative real-time-PCR (RT-qPCR)

The reverse transcription was performed using HiScript II Q RT SuperMix for qPCR Kit (Vazyme, cat#R223-01), and quantitative PCR was performed using ChamQ SYBR qPCR Master Mix (Vazyme, cat#Q321-02) according to the manufacturer’s protocol. The conditions for the reactions were as follows: 95 °C for 30 s, 95 °C for 10 s and 60 °C for 30 s for 40 cycles. Gene expression was normalized to GAPDH mRNA content, and the relative expression of target gene was determined from replicate samples using the 2^−ΔΔCt^ (Ct, cycle threshold). Primer sequences were as follows: GAPDH: 5′-TCATTGACCTCAACTACATGGTTT-3′ (sense) and 5′-GAAGATGGTGATGGGATTTC-3′ (antisense); BVES: 5′-ACCAGCGAGCCTCTGCCAAGA-3′ (sense) and 5′-CCTCACTTCCTCCCTCCGACTCT-3′ (antisense).

### Plasmids

The stable cell lines Huh7-shBVES, SK-hep-1-BVES, and SMMC-7721-BVES were constructed using plasmids transfection. Plasmid vectors encoding the human BVES short hairpin RNAs (shRNA) (shBVES) and BVES expression plasmids (BVES) were obtained from Genechem (Shanghai, China). The sequences were listed as follow: shBVES: 5′-CCTCCAGATTTGTTCAGAA-3′. For BVES expression plasmid construction, the open reading frame of BVES (NM_147147) was cloned into the plasmid vector GV141. The method of BVES plasmid lentiviral transfection was performed as described previously [18].

### Co-immunoprecipitation (Co-IP) analysis

Cells were lysed on the ice with NP-40 solution for 40 min. After centrifugation, supernatants were mixed with Protein A/G Agarose Beads (Abmart, cat#HY-K0202) and appropriate antibodies BVES (Santa cruz, cat#sc-374081), Normal Mouse IgG (Abmart, cat#2729) overnight at 4 °C. The agarose beads were separated from the mixture, and incubated with the supernatants. Precipitated proteins were washed with lysis buffer four times. Samples were collected for subsequent western blotting analyses.

### In vivo metastasis analysis

All animal experimental procedures were conducted following National Institutes of Health Guide for the Care and Use of Laboratory Animals. The protocol was approved by the Committee of Ethics of Animal Experiments of Tongji Hospital, Huazhong University of Science and Technology. Six-week-old BALB/C male nude mice were raised in specific pathogen-free (SPF) conditions. Firstly, 5 × 10^6^ luciferase labeled HCC cells were injected subcutaneously into the mice to establish a subcutaneous xenograft tumor model. When the subcutaneous tumor of the nude mice grew to 1 cm^3^ in size, the tumor was resected and cut into small tumors of 1 mm^3^ in size. Eight-week-old nude mice were randomly divided into SMMC-7721-BVES-luc group and SMMC-7721-Control-luc group, with 10 mice in each group. After anesthesia, the isolated small tumor tissues were placed into the liver lobes of the mice. Tumor metastasis was monitored using the bioluminescence every 2 weeks. For in vivo signals detection, d-luciferin at 15 mg/kg was injected intraperitonially into the nude mice. After 8 weeks, the mice were sacrificed, liver and lung tissues were resected and collected for further histological examination.

### RhoA activity analysis

The RhoA activity was analyzed by a RhoA activation assay kit (Wuhan Newstead Biotechnology, cat# 80601), and the operating procedure was performed according to the instructions. In brief, a murine monoclonal antibody that specifically recognizes the RhoA-activated conformation could specifically bind to RhoA-GTP active protein in cell lysates. Then, Protein A/G was used to adsorb the antigen–antibody conjugate, following by detection through western blotting analyses using a rabbit polyclonal antibody that specifically recognized RhoA. Moreover, before conjugation by Protein A/G, a fraction of cell lysates of each sample was used to detect total RhoA, ZO-1, GEFT, BVES and GAPDH protein expression level as described in section of Western blot analyses.

### Statistical analysis

Student’s *t*-test was used for quantitative data analysis, and Fisher’s exact test was utilized to analyze categorical data. Two-sided *P* < 0.05 was considered statistically significant. Statistical analyses were calculated using SPSS software (version 21.0) and Prism 6.0 (GraphPad Software).

## Results

### Observation of extrusion in HCC

**Fig. 2:**
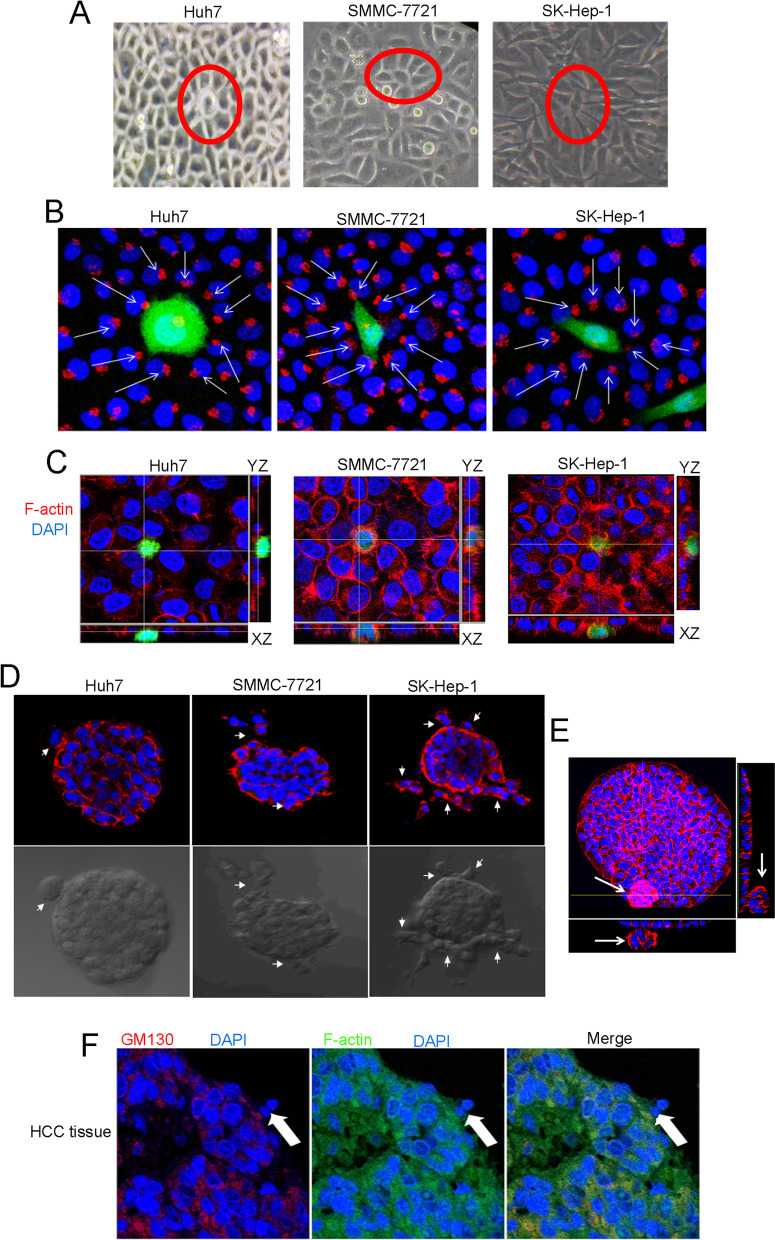
Extrusion in HCC cells and tissues. **A** The representative images of extrusion in Huh7, SMMC-7721, and SK-Hep-1 cells (× 200). Certain cells were surrounded and suffered “collective attack” by neighboring cells. **B** Golgi matrix protein GM130 (the arrows) reflected the direction of force and migration of cells (× 200). Red, GM130; blue, nuclei; green, GFP labeled HCC cells. **C** Confocal projections analysis of HCC cells extrusion by overlaying the XY plane of the actin-extruding ring with the XY plane of the DNA of the extruded cells. Red, F-actin; blue, nuclei; green, GFP labeled HCC cells. **D** 3D cell culture model for detecting HCC cells extrusion (× 200). Red, F-actin; blue, nuclei. Arrows pointed to extruded cells. The pictures below were the corresponding images of the cells under microscope. **E** Confocal projections analysis of Huh7 cells extrusion in 3D cell culture model. **F** The representative images of extrusion in HCC tissues (× 200). Red, GM130; blue, nuclei; green, F-actin. Arrows pointed to extruded cells

The cell extrusion phenomenon in epithelial cells has been observed and reported in previous studies, such as apoptotic cell extrusion and transformed cell extrusion [19]. However, few studies focused on cancer cell extrusion, especially in HCC. As shown in Fig. 2A, in confluent monolayers of cultured HCC cells, certain cells were surrounded by neighboring cells, suffering a “collective attack”, the attacked cells then got round, which was consistent with previous studies on cell extrusion [20, 21]. Since the location of Golgi complex reflects cell movement direction, HCC cells labeled with green fluorescent protein (GFP) were co-cultured with HL7702 cells at ratio of 1:100, and then golgi matrix protein 130 (GM130) staining was used to detect the direction of force and migration of neighboring cells. Excitingly, the results showed that both the force and migration directions in neighboring cells were directed towards the HCC cells, seemingly trying to squeeze them out of the monolayer (Fig. 2B). F-actin is a type of actin cytoskeleton, which plays an important role in cell migration. Previous studies have revealed that the F-actin flow and actin ring is involved in the process of epithelial extrusion driven by multiple signaling [22–24]. Here, we also utilized confocal projections with F-actin staining to assess cancer cell extrusion. After overlaid the XY plane of the actin-extruding ring with the XY plane of the DNA of the extruded cell, as shown in XZ and YZ cross-sections, the nuclei of extruding cells were obviously not in the same plane of that in the nuclei of the neighboring cells, suggesting that certain HCC cells were being extruded by surrounding HL7702 cells (Fig. 2C). To mimic an in vivo environment as closely as possible, cells were planted and grown in 3D cell culture models to form overcrowded cell mass. As shown in Fig. 2D, several cells were found to be extruded from the mass in Huh7, SMMC-7721 and SK-Hep-1 HCC cells, and F-actin staining was obviously higher in the extruding cells and their neighbors. The 3D imaging vividly showed the extrusion Huh7 cells from the cell mass (Fig. 2E). Furthermore, HCC cell extrusion could also be observed in tumor tissues from HCC patients (Fig. 2F).

### BVES expression was decreased in HCC tissues and cells extruded

**Fig. 3:**
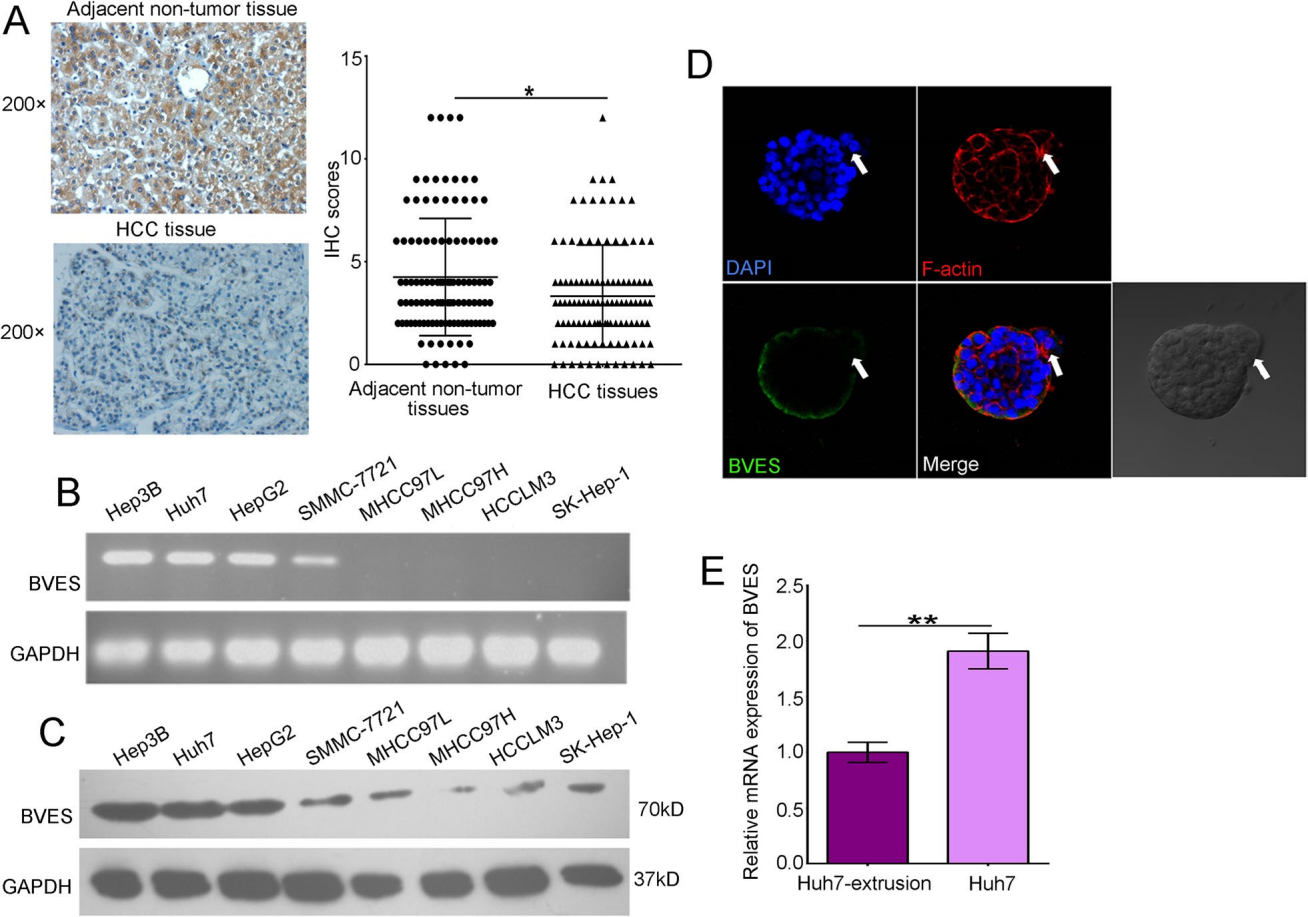
BVES expression was decreased in HCC tissues and cells extruded. **A** The representative IHC images of BVES in adjacent nontumor and HCC tissue (left); IHC scores of BVES staining in human samples (right). **B** PCR analysis showing the mRNA expression level of BVES in HCC cell lines. **C** Western blotting assays showing the protein expression of BVES in HCC cell lines. **D** IF confocal detection of cellular BVES expression in 3D cell culture model. The arrow pointed to a cell extruded. Red, F-actin; blue, nuclei; green, BVES; and the last was the corresponding images of the cell mass under microscope. **E** The mRNA expression of BVES in Huh7 and Huh7-extrusion cells. Bars represent mean ± S.D. of three independent experiments, *P < 0.05, **P < 0.01

We have reported that BVES expression was decreased in HCC tissues, and inhibition of BVES may trigger HCC migration previously [25]. However, the exact role of BVES in HCC metastasis still remains unknown. In the present study, we further analyzed the expression of BVES in 108 pairs of HCC tissues and corresponding adjacent nontumor tissues. Consistent with the previous study, the results showed that BVES expression was lower in HCC tissues than in corresponding adjacent nontumor tissues (Fig. 3A). Moreover, we also analyzed the mRNA and protein expression level of BVES in HCC cell lines. The results showed that BVES expression level was lower in cells with relatively high shedding ability, while in those with low shedding ability, BVES expression level was much higher (Fig. 3B, C). Since the expression level of BVES in different HCC cell lines was inversely correlated with cell shedding ability [15], then we analyzed the expression of BVES in the cells extruded. The results of IF and RT-qPCR showed that BVES was decreased in cells extruded than those in situ (Fig. 3D, E). These data suggested that BVES might be involved in HCC cell extrusion.

### BVES downregulation enhanced HCC cells extrusion

**Fig. 4:**
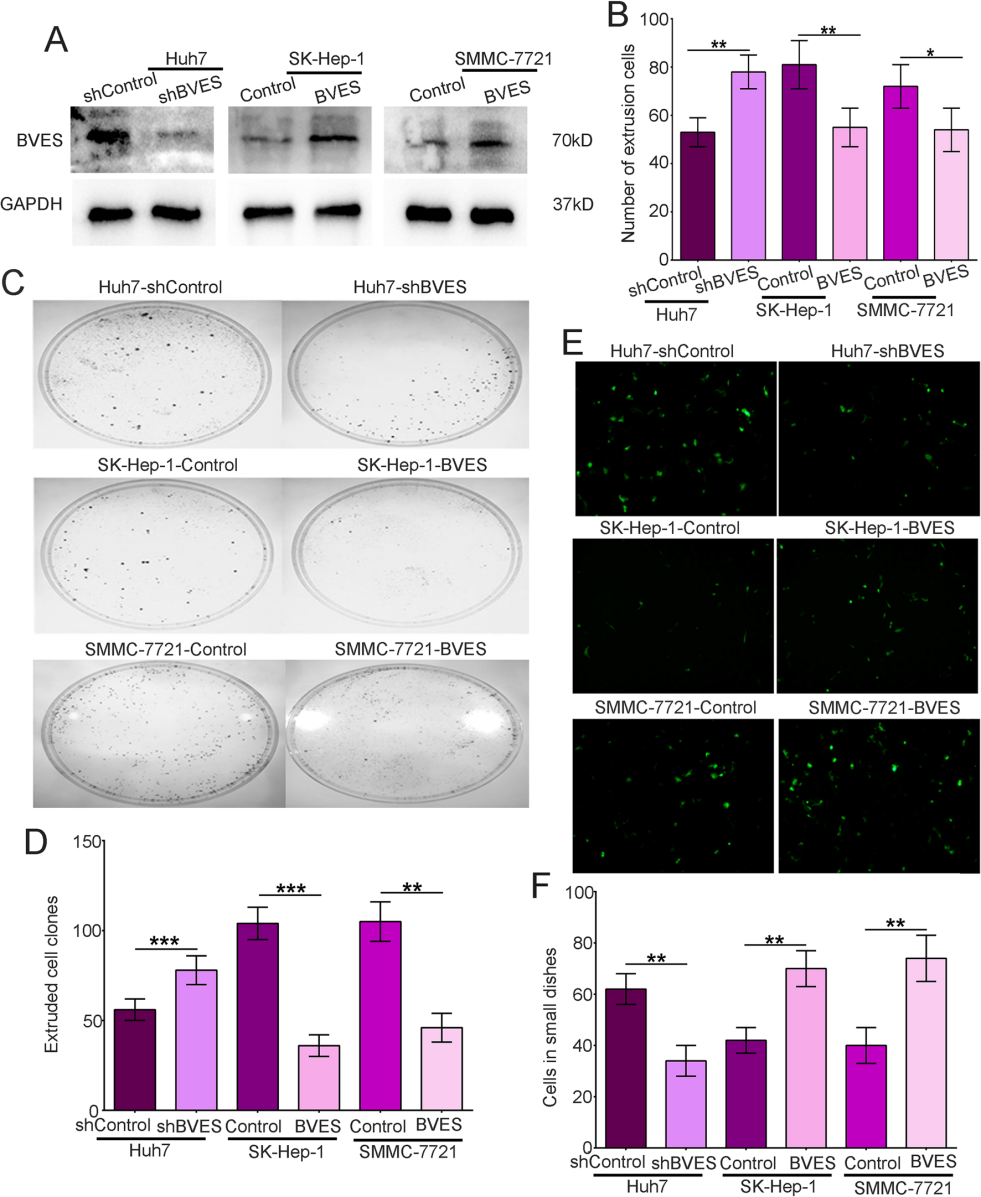
BVES downregulation enhanced HCC cells extrusion. **A** Western blot analysis of the expression of BVES after plasmids transfection. **B** The number of extrusion cells of Huh7 cells with BVES knockdown and SK-Hep-1 cells/SMMC-7721 with BVES overexpression in the silicone chamber model. **C** Representative images of cell clones in the large culture dish of extruded indicated cells in the petri dish inversion model. **D** The clone numbers of extruded indicated cells in the large culture dish of extruded indicated cells in the petri dish inversion model. **E** GFP-labeled HCC cells were mixed with HL7702 cells at a ratio of 1:100 to grow to confluence in the petri dish inversion model. The number of cells remained in the small dish inversely correlated with the number of extruded HCC cells (× 40). Representative images of the remaining indicated HCC cells in the small dish per field of vision. **F** The number of remaining indicated HCC cells in the small dish per field of vision. Bars represent mean ± S.D. of three independent experiments, *P < 0.05, **P < 0.01, ***P < 0.001

To demonstrate the role of BVES in the extrusion of HCC cells, we established three stable cell lines Huh7-shBVES, SK-hep-1-BVES, and SMMC-7721-BVES using plasmids transfection (Fig. 4A). The results of silicone chamber model showed that BVES downregulation significantly enhanced extrusion capacities of Huh7 cells (low initial metastatic potential), whereas upregulation of BVES decreased SK-Hep-1 and SMMC-7721 cells extrusion (high initial metastatic potential) (Fig. 4B). We have reported an in vitro petri dish inversion model to detect crowding-induced cancer cells extrusion previously [15], and we further utilized this model to determine the role of BVES in HCC cells extrusion. The results suggested that BVES knockdown increased extruded cell clones in large dishes and decreased the number of cells in small dishes, whilst there were less extruded cell clones and more cells remained in small dished amongst those with BVES overexpression (Fig. 4C–F).

**Fig. 5:**
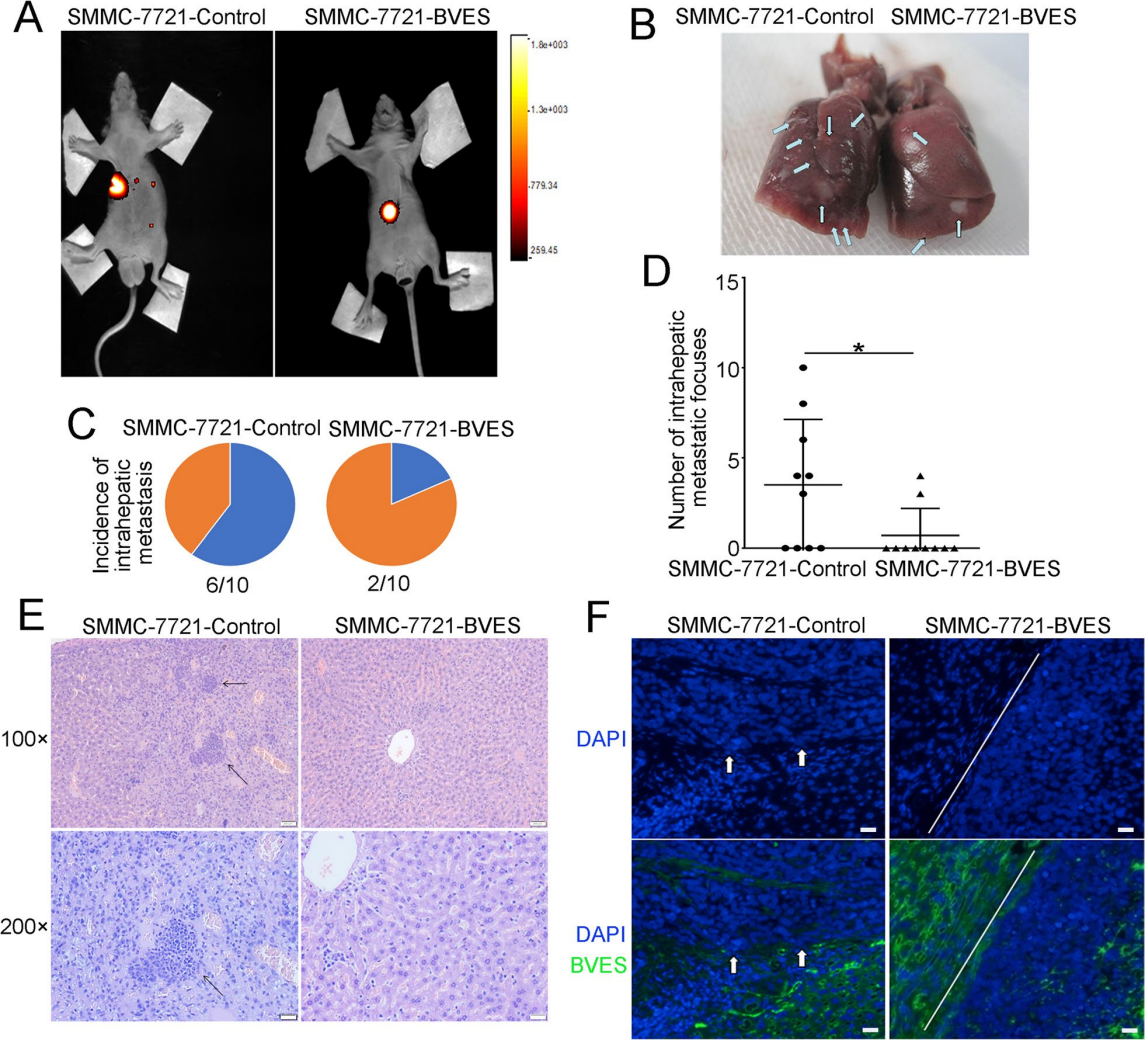
BVES overexpression inhibited HCC metastasis in vivo. **A** Representative bioluminescent images of mice in SMMC-7721-Control and SMMC-7721-BVES groups. **B** Representative liver images of mice in SMMC-7721-Control and SMMC-7721-BVES groups. **C** The incidence of intrahepatic metastasis in SMMC-7721-Control and SMMC-7721-BVES groups. **D** The number of intrahepatic metastatic focuses in SMMC-7721-Control and SMMC-7721-BVES groups. **E** Representative H&E staining images of liver tissues of mice in SMMC-7721-Control and SMMC-7721-BVES groups [scale bars: 200 μm (upper); 100 μm (lower)]. **F** Representative IF staining images of intrahepatic peri-tumor cell extrusion. The left side of the SMMC-7721-BVES picture was the adjacent nontumor tissues and capsule of nude mice, and the right side was the transplanted tumor in situ. The upper part of the SMMC-7721-Control picture was the orthotopic tumor of the transplanted tumor, and the lower part was the adjacent nontumor tissues and capsule of nude mice. Arrows indicate partially extruded cells. Blue, nuclei; green, BVES (scale bars: 200 μm). Bars represent mean ± S.D., *P < 0.05

To further investigate the role of BVES in tumor metastasis in vivo, cells were transplanted into livers of nude mice. Representative images of bioluminescent imaging (BLI) and livers were shown in Fig. 5A and B. In the SMMC-7721-BVES group, only 2 mice developed intrahepatic metastasis presenting with multi-metastatic focuses, while 6 mice developed intrahepatic metastasis in the control group (Fig. 5C, D). The representative histological images were shown in Fig. 5E. Additionally, the IF staining results showed that extrusion in the tumor border was reduced in SMMC-7721-BVES group than that in the control group, presenting as orderly tumor border in SMMC-7721-BVES mice and irregular tumor border in the control group (Fig. 5F). These results suggested that BVES decreased HCC cells extrusion, thus leading to the inhibition of HCC metastasis.

### BVES regulated RhoA activity by co-localization with ZO-1 and GEFT

**Fig. 6:**
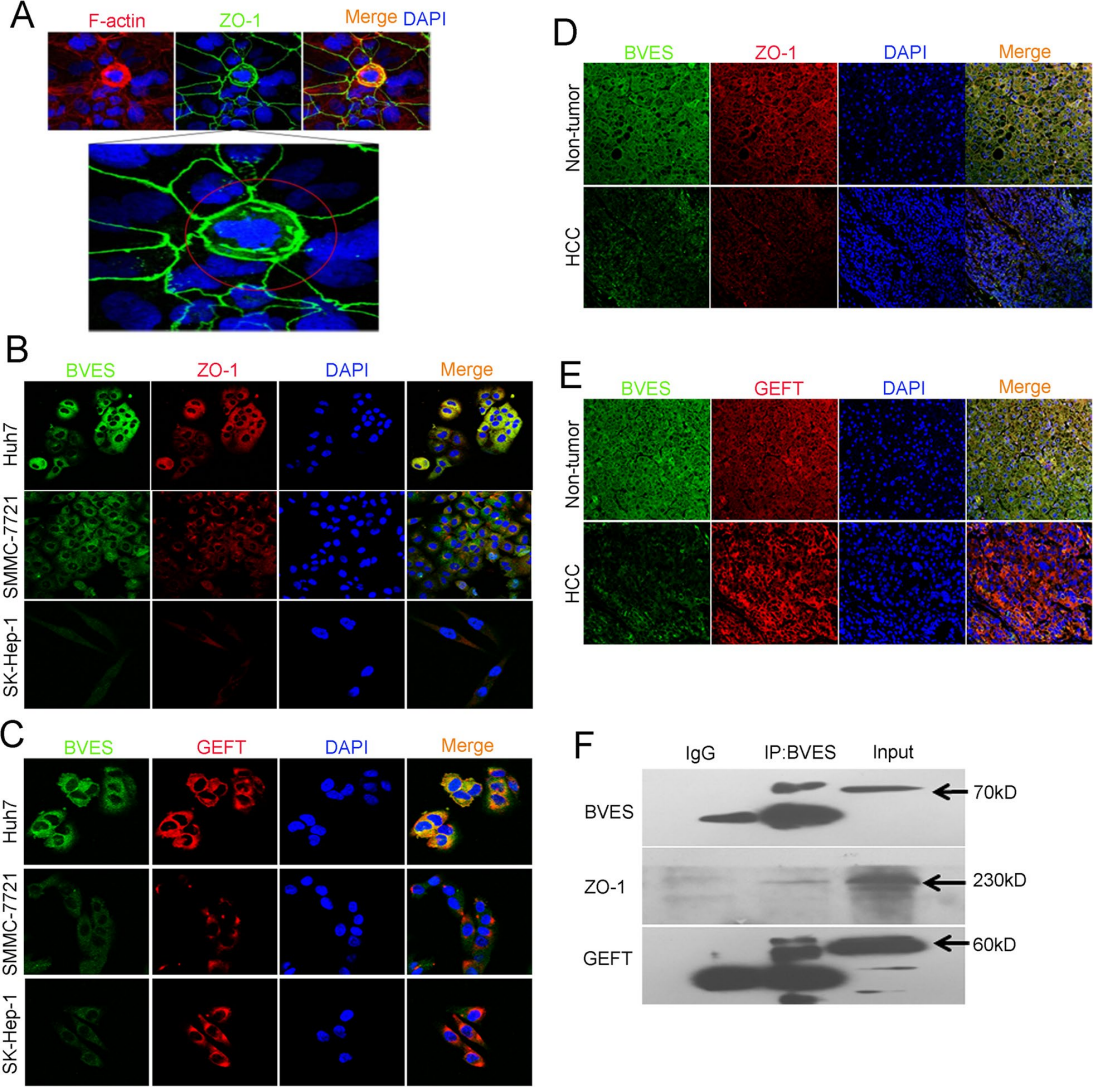
BVES co-localized with ZO-1 and GEFT. **A** The representative IF staining images of extruded Huh7 cells. Red, F-actin; blue, nuclei; green, ZO-1. **B** IF staining showing expression and co-localization of BVES and ZO-1 in HCC cell lines. Red, ZO-1; blue, nuclei; green, BVES. **C** IF staining showing expression and co-localization of BVES and GEFT in HCC cell lines. Red, GEFT; blue, nuclei; green, BVES. **D** IF staining showing expression and co-localization of BVES and ZO-1 in HCC and adjacent nontumor tissues. Red, ZO-1; blue, nuclei; green, BVES. **E** IF staining showing expression and co-localization of BVES and GEFT in HCC and adjacent nontumor tissues. Red, GEFT; blue, nuclei; green, BVES. **F** BVES protein was immunoprecipitated from Huh7 cells and subjected to western blotting assays to detect its interaction with ZO-1 and GEFT. IgG was used as a negative control

**Fig. 7:**
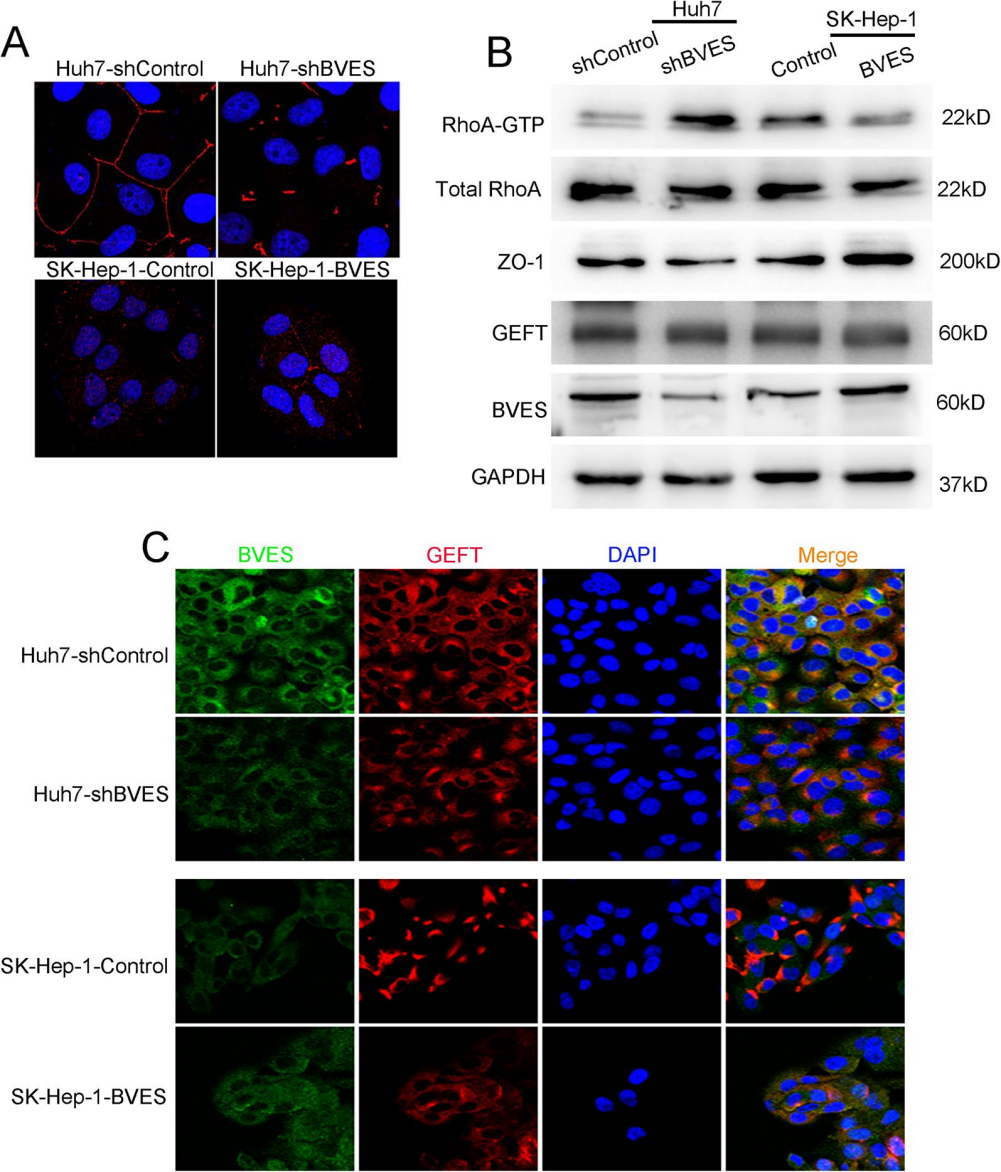
BVES influenced RhoA activity by regulating ZO-1 and GEFT. **A** IF staining showing expression and subcellular localization of ZO-1 in Huh7 cells with BVES knockdown and SK-Hep-1 cells with BVES overexpression. Red, ZO-1; blue, nuclei. **B** Pull-down and western blotting assays showing the RhoA activity and ZO-1 expression in Huh7 cells with BVES knockdown and SK-Hep-1 cells with BVES overexpression. **C** IF staining showing the subcellular localization of ZO-1 in Huh7 cells with BVES knockdown and SK-Hep-1 cells with BVES overexpression. Red, ZO-1; blue, nuclei; green, BVES

RhoA-mediated assembly and contraction of an intercellular actomyosin ring is the primary mechanism of both apical and basal extrusion. BVES has been reported to regulate RhoA activation in TM cells by sequestering GEF-H1 with BVES/ZO-1 complex in the cell membrane, but it was controversial in some cells recently. We have demonstrated that the expression and co-localization of BVES and ZO-1 was decreased upon HCC cells metastasis [18]. In this work, we found that ZO-1 was redistributed in extruded Huh7 cells by IF assays, and the expression of ZO-1 was increased in the cytoplasm of extruded HCC cells, meanwhile, F-actin staining intensity between the extruded cells and neighboring cells was significantly enhanced, suggesting that the redistribution of ZO-1 might be associated with the extrusion of HCC cells (Fig. 6A). As shown in Fig. 6B and C, in Huh7 cells with low metastatic potential, BVES co-localized with ZO-1 and GEFT mainly on the cell membrane. Interestingly, in SK-Hep-1 cells with high metastatic potential, the expression of BVES and ZO-1 were decreased mainly on the cell membrane, while the expression of GEFT in the cytoplasm increased. Similarly, the results of IF assays of human tissues showed that BVES was co-localized with ZO-1 and GEFT in nontumor tissues, and these three were mainly expressed on the cell membrane. Nevertheless, in HCC tissues, the membrane expression BVES and ZO-1 were significantly decreased in spite of the higher cytoplasmic expression of GEFT (Fig. 6D, E). In addition, the results of Co-IP assays further confirmed the colocalization of BVES and ZO-1 or GEFT (Fig. 6F). We have observed that ZO-1 might participate in the extrusion of HCC cells and co-localize with BVES, we wondered whether BVES was involved in ZO-1 regulation. By IF staining analysis, we found that BVES knockdown resulted in the reduced the expression and localization of ZO-1 on the cell membrane, which had an impact on cell tight junctions, while BVES overexpression showed an opposite effect (Fig. 7A). Moreover, the results of pull-down analysis showed that downregulation of BVES increased RhoA activity, and upregulation of BVES in SK-Hep-1 cells decreased RhoA activity. Meanwhile, the western blotting analysis results indicated that the expression of ZO-1 was decreased in cells with BVES knockdown and ZO-1 expression was upregulated in cells with BVES overexpression (Fig. 7B). GEFT is an important regulator of Rho protein activity, responsible for converting Rho-GDP to active Rho-GTP, and its cellular sub-localization was correlated with its function. Through IF assays, we observed that GEFT was diffusely distributed in the cytoplasm in Huh7 cells and partially co-localized with BVES on the cell membrane, whereas after downregulation of BVES expression, GEFT accumulated in local cytoplasmic sites, which might be associated with its functions in cells. However, upregulation of BVES in SK-Hep-1 cells led to an opposite result (Fig. 7C). These results suggested that BVES not only co-localized with ZO-1 and GEFT, but also regulated their expression and location, thus affecting RhoA activity in HCC cells, which might be the underlying mechanism of BVES regulated HCC cells extrusion.

## Discussion

Classical theories suggest that the maintenance of epithelial layer homeostasis due to the division of epithelial cells to replace apoptotic cells, which in turn maintains a stable number of epithelial cells [26, 27]. However, several recent studies have reported that in the epithelial layer, the number of local cells increases, and when the number of cells increases to 1.6–1.8 times as the normal, some cells have reduced connections with surrounding cells, as well as with the extracellular matrix. Then, these cells will be extruded by neighboring cells through mechanical force, and the extruded cells will undergo anoikis because of the lack of survival signals [28–30]. Some tumor cells themselves can resist anoikis by upregulating survival signals [31]. We speculate that if tumor cells of epithelial origin are extruded, they may continue to survive and even metastasize. It has been suggested genetically mutated cells extruded apically might be eliminated or form carcinoma in situ, while tumor cells extruded basally have potentials to break through the basal layer and then metastasize to other organs [32]. Hepatocytes are a kind of epithelial cells, but their structure is distinct from other epithelia in that there is no basement membrane spacing between the hepatocytes epithelium and the hepatic blood sinusoids [33]. On the one hand, HCC cells extruded apically could cause hematogenous metastasis. On the other hand, basally extruded HCC cells might invade basement membrane and potentially initiate lymphatic metastasis [8]. Therefore, extrusion of HCC cells will probably not be strictly directional, and once extruded, most of them can survive and metastasize, which might also be a reason why HCC is prone to metastasis in the early stage.

In order to verify the above speculation, first, we established silicone chamber and petri dish inversion models to detect the extrusion of HCC cells, and observed that the HCC cells could be extruded by both normal hepatocytes and neighboring HCC tumor cells. The extruded HCC cells could continue to survive and form new cell clones. To visualize the effect of mechanical force during cell extrusion, we stained the cells with F-actin and GM130 staining, and the results showed that F-actin staining intensity between the extruded cells and neighboring cells was obviously enhanced, while the direction of the forces of surrounding cells were all directed towards the extruded cells. In addition, we also noticed the interesting phenomenon in 3D cell culture and human HCC tissue specimens. These results revealed the existence of cancer cell extrusion in HCC, which might be involved in HCC metastasis.

Previous studies have demonstrated that live cell extrusion induced by crowding is always associated with activation of mechanosensitive PIEZO1 channels, and PIEZO1 may extrude excess live cells along the epithelium apical by activating S1P/S1P2 signaling pathway [4]. However, increasing evidence has suggested extrusion of tumor cells might be different from apoptotic and living cells. Jody Rosenblat et al. have reported that oncogenic K-Ras cells downregulate both S1P and its receptor S1P2 to inhibit apical extrusion, thus promoting basal extrusion [20]. Several other potential mechanisms of tumor cell extrusion also have been noticed. Molecules that control epithelial cell polarity may regulate the direction of cancer cell extrusion by altering cell polarity [34, 35]. Hyperactivation of Rho proteins can be also involved in regulation of tumor cell extrusion in that Rho proteins activation leads to activation of F-actin and myosin at intercellular junction sites, thus triggering cell contraction, which has an influence on cell extrusion [36–38]. Moreover, weakened adhesion between cells and between cells and extracellular matrix might result in increased tendency to shed of some cells [39, 40]. Nevertheless, the regulation and mechanisms of tumor cell extrusion remain largely unclear and require further investigations.

We have demonstrated BVES inhibition as an early event in the metastasis of HCC [25], here, we also focus on the involvement of BVES in the HCC cell extrusion, especially in the metastasis of HCC. Consistent with our previous study, we found that the adhesion molecule BVES expression was reduced in HCC and it might be involved in early metastasis of HCC [25]. In the cell extrusion model, BVES expression was decreased in extruded cells and neighboring cells. The results of further explorations showed that alteration of BVES expression had an effect on the number of HCC cells extrusion. The orthotopic xenograft model results demonstrated that BVES overexpression could inhibit the intrahepatic and extrahepatic metastasis of HCC cells, and the inhibition of extrusion of neighboring tumor cells around the in situ tumor might be part of the mechanisms. We also investigated the potential mechanisms by which BVES inhibited extrusion of HCC cells. The present study results have shown that BVES co-localized with ZO-1 and GEFT to regulate tumor cell extrusion by reducing RhoA protein activity, thus inhibiting myosin contraction. More studies are needed to elucidate the role and mechanisms of BVES-regulated HCC cell extrusion.

Although we have observed that BVES regulated tumor cells extrusion in HCC through in vitro experiments and animal models, the role of tumor cell extrusion in HCC metastasis has not been directly observed so far. Observation of tumor cell extrusion by live imaging and microscopic techniques in zebrafish model might be the focus of our future research.

In addition, cell migration and invasion are most important process of HCC cell metastasis. Unlike the theories of cell migration and invasion, which focus on the increase of metastatic ability of tumor cells themselves, the theory of cancer cell extrusion emphasizes the joint action of extruded cells and neighboring cells, and the intercellular mechanical forces are the key to cancer cell extrusion. The extent of the role of cancer cell extrusion mechanism in HCC metastasis and whether there is any intersection between it and other metastasis mechanisms need to be further investigated.

## Conclusions

In conclusion, the present study has observed that in overcrowding, HCC cells can be extruded by adjacent hepatocytes or tumor cells, and then continue to grow and metastasis. BVES overexpression inhibited HCC cells extrusion and metastasis through BVES/ZO-1/Rho GTPases pathway. Our study revealed the role and potential mechanisms of BVES-regulated tumor cells extrusion in HCC metastasis, contributing to a more comprehensive understanding of HCC metastasis, and provided a promising target for developing novel HCC therapy strategies.

## Data Availability

The datasets analyzed for this study can be accessable from the corresponding author on reasonable request.

## Abbreviations

HCC: Hepatocellular carcinoma
BVES: Blood vessel epicardial substance
ZO-1: Zonula occludens-1
GEF: Guanine nucleotide exchange factor
TM: Trabecular meshwork
TJ: Tight junction
3D: Three-dimensional
IF: Immunofluorescence
BLI: Bioluminescent imaging
RhoA: Ras homolog family member A
GFP: Green fluorescent protein
GM130: Golgi matrix protein
S1P: Sphingosine-1-phosphate
